# Comparison of the Effect of Pyridostigmine and Sugammadex on Emergence Delirium in Pediatric Patients Undergoing Strabismus Surgery: A Randomized Controlled Trial

**DOI:** 10.3390/jcm15155855

**Published:** 2026-07-27

**Authors:** Chung-Sik Oh, Hyun Jin Shin, Seon-Ju Park, Yea-Ji Lee

**Affiliations:** 1 Department of Anesthesiology and Pain Medicine, Konkuk University Medical Center, Seoul 05030, Republic of Korea; ohcsik@naver.com (C.-S.O.); tjswn9410@naver.com (S.-J.P.); 2 Research Institute of Medical Science, Konkuk University School of Medicine, Seoul 05030, Republic of Korea; shineye@kuh.ac.kr; 3 Institution of Biomedical Sciences and Technology, Konkuk University School of Medicine, Seoul 05030, Republic of Korea; 4 Department of Ophthalmology, Konkuk University Medical Center, Seoul 05030, Republic of Korea

**Keywords:** anesthesia, general, emergence delirium, neuromuscular blockade, pediatrics, strabismus, surgical procedure, ophthalmic

## Abstract

**Background**: Emergence delirium (ED) is a common complication in pediatric patients following general anesthesia. Reduced cholinergic neurotransmission is closely associated with cognitive impairment and an increased risk of delirium. This study aimed to compare the effects of a cholinesterase inhibitor versus sugammadex–used as neuromuscular blockade reversal agents–on the incidence of ED following pediatric strabismus surgery. **Methods**: Seventy-six patients, aged 4–7 years, were enrolled and randomly allocated to either the pyridostigmine group (Group P) or the sugammadex group (Group S). The dose of each agent was determined according to the train-of-four count or ratio. The Pediatric Anesthesia Emergence Delirium (PAED) scale was assessed at 15-min intervals in the post-anesthesia care unit until the score fell below 10. ED was defined as a PAED score ≥ 10. **Results**: Of the 73 patients who completed the study, 36 were in Group P, and 37 were in Group S. The incidence of ED was 50.0% in Group P and 40.5% in Group S (odds ratio = 0.682; 95% confidence interval = 0.270–1.721; *p* = 0.417). The peak PAED scores were 10.0 (7.0, 15.5) in Group P and 9.0 (7.5, 11.5) in Group S (median [interquartile range]; *p* = 0.277). **Conclusions**: The choice of neuromuscular reversal agent did not significantly affect the incidence of ED in pediatric patients undergoing strabismus surgery.

## 1. Introduction

Emergence delirium (ED) is a well-known postoperative complication of pediatric anesthesia. It is characterized by altered consciousness, disorientation, and psychomotor agitation, with affected children being inconsolable, irritable, uncooperative, and prone to crying, moaning, or incoherent behavior [1,2,3]. Although the etiology of ED remains elusive, several predisposing factors have been identified, including rapid recovery from general anesthesia, the use of inhalational anesthetics, inadequate postoperative pain management, and agitation during induction. Inhalational agents, such as sevoflurane and desflurane, are commonly used in pediatric anesthesia because they provide rapid, smooth induction, quick emergence, hemodynamic stability, and amnesia [4]. Although their rapid recovery from anesthesia is attributed to their lower blood solubility compared to halothane, this abrupt washout and rapid awakening have been shown to contribute to the development of ED [5]. Additional risk factors comprise the surgical site (specifically ophthalmic and otorhinolaryngological surgeries), airway obstruction, and temperature dysregulation such as hyper- or hypothermia [5,6,7,8]. Notably, recent studies have reported that intraoperative hypothermia is associated with an increased risk of ED, and this relationship is potentially linked to hypothermia-induced electroencephalogram alterations, specifically burst suppression [9,10,11]. ED is highly prevalent among preschool-aged children; its reported incidence ranges widely from 10% to 80% depending on the definition adopted, patient demographics, surgical characteristics, assessment methods, and monitoring periods used in each study [12,13,14].

Cholinesterase inhibitor (ChEI) is a conventional agent for the reversal of neuromuscular blockade (NMB) by preventing the cleavage of acetylcholine within the synaptic cleft in the neuromuscular junction. However, ChEI has to be co-administered with anticholinergic agents such as atropine or glycopyrrolate to prevent muscarinic side effects. Cholinergic dysregulation induces delirium by allowing irrelevant intrinsic and sensory data to permeate conscious awareness. Consequently, mitigating serum anticholinergic activity is essential for the resolution of delirium [15,16,17]. The American Geriatrics Society Beers criteria list ChEIs and related anticholinergic agents to be avoided in elderly patients because their use is associated with an increased anticholinergic burden, which may elevate the risk of cognitive impairment and delirium [18].

Sugammadex is a modified gamma-cyclodextrin that reverses NMB by encapsulating free steroidal, nondepolarizing neuromuscular blocking agents, specifically rocuronium and vecuronium, in the plasma. As it does not inhibit acetylcholinesterase, it eliminates the need for concomitant anticholinergic administration, thereby facilitating rapid NMB reversal and minimizing residual NMB without inducing cholinergic side effects [19].

While both sugammadex and conventional ChEIs are widely used for the reversal of NMB in pediatric patients, few studies have evaluated their comparative effects on ED in this population. In addition, previous research has indicated that NMB is associated with an increased incidence of ED [20]. Therefore, we hypothesized that the incidence of ED would be higher in patients receiving ChEI compared to those receiving sugammadex.

## 2. Materials and Methods

This prospective, randomized, controlled, double-blind trial was approved by the Institutional Review Board (IRB) of Konkuk University Medical Center (KUMC2023-05-082) and registered at clinicaltrials.gov (NCT06035757, Principal investigator: Y.-J.L., Registration date: 13 September 2023). The study complied with the Declaration of Helsinki. Verbal assent was obtained from the patients, and written informed consent was provided by their legal representatives (parents).

### 2.1. Study Subjects

Eligible participants were pediatric patients aged 4–7 years with an American Society of Anesthesiologists (ASA) physical status of I or II undergoing elective strabismus surgery. The exclusion criteria were ASA physical status ≥ III, hepatic or renal impairment, underlying neuromuscular disease, cardiomyopathies, arrhythmias, a history of hypersensitivity or anaphylaxis to the study medication, or refusal to participate.

Patients were randomly assigned to either Group P (pyridostigmine) or Group S (sugammadex). An independent researcher, not involved in clinical care or assessment, performed block randomization (block size of 4) using Random Allocation Software (version 1.0.0; Mahmood Saghaei, Isfahan University of Medical Sciences, Isfahan, Iran). Patients, legal representatives, and outcome assessors remained blinded to group allocation throughout the study.

### 2.2. Anesthetic Procedures

Patients entered the operating room (OR) accompanied by a parent or caregiver. Routine monitoring included non-invasive blood pressure, pulse oximetry, electrocardiogram, bispectral index (BIS), and NMB monitoring via the train-of-four (TOF) stimulation modality. The level of NMB was measured using the NMT MechanoSensor™ (GE HealthCare Technologies Inc., Chicago, IL, USA). Preoxygenation was performed via spontaneous mask ventilation with an inspired oxygen fraction of 0.8, followed by intravenous propofol 2 mg/kg. Once consciousness was lost, parents or caregivers left the OR. Manual mask ventilation was performed with 80% oxygen and sevoflurane. After calibration with a supramaximal current, a reliable baseline TOF ratio was established. When the TOF ratio had stabilized at 100%, intravenous rocuronium 0.6 mg/kg was administered. Tracheal intubation was then performed when the TOF count reached 0. Anesthesia was maintained with sevoflurane and a continuous infusion of remifentanil. The sevoflurane concentration was titrated to maintain a BIS of 40–60, while the remifentanil dose was adjusted to maintain hemodynamic stability within ±20% of baseline, not exceeding 0.25 μg/kg/min. At the end of the procedure, NMB reversal was performed with either pyridostigmine or sugammadex based on the indicated TOF count or ratio. In Group P, intravenous pyridostigmine 350 μg/kg was administered for a TOF count of 2–3 or a TOF ratio < 0.4, and 200 μg/kg for a TOF ratio ≥ 0.4. Glycopyrrolate and pyridostigmine were administered in a 1:20 ratio (0.05 mg glycopyrrolate per 1 mg pyridostigmine) [21]. If the TOF count was <2, reversal was postponed until the TOF count was reached 2. In Group S, intravenous sugammadex 2 mg/kg was given for a TOF count ≥ 2, and 4 mg/kg for a post-tetanic count (PTC) ≥ 1 to a TOF count of 1. If the PTC was <1, reversal was delayed until it reached ≥ 1. All dosages followed established guidelines [22,23]. Tracheal extubation was performed once the TOF ratio reached ≥ 0.9. The TOF count or ratio was recorded at the time of reversal and extubation.

### 2.3. Assessment of ED

When the Ramsay Sedation Scale score reached ≤ 3, patients were transferred to the post-anesthesia care unit (PACU). Blinded assessors evaluated ED using the Pediatric Anesthesia Emergence Delirium (PAED) scale (Table 1) [24]; to minimize subjectivity and inter-rater variability [25], they had completed comprehensive training before the study began. PAED scores were recorded at 15 min intervals: upon PACU arrival (T1), and 15- and 30 min post-arrival (T2 and T3, respectively). PAED score ≥ 10 was considered diagnostic of ED [12,26], prompting the administration of intravenous fentanyl 0.5 μg/kg (up to a maximum of 2 μg/kg) as a rescue medication.

The primary endpoint was the incidence of ED in the PACU. Secondary endpoints included the peak PAED score and the additional requirement for rescue fentanyl doses.

### 2.4. Sample Size Calculation and Statistical Analysis

Sample size estimation was based on a prior study [12], assuming an ED incidence of 72% in the control group and 36% in the intervention group. To achieve 80% power with a two-tailed α error of 0.05, 33 patients per group (66 in total) were required for the primary analysis. Considering an anticipated attrition rate of 15% and our IRB’s recommendation to minimize the enrollment of pediatric participants, we planned to enroll a total of 76 patients.

Continuous variables are expressed as mean ± standard deviation (SD), median (interquartile range), and categorical variables as number (%), as appropriate. Normality was evaluated with the Shapiro–Wilk test. Intergroup differences in PAED scores were analyzed using repeated measures ANOVA (RM ANOVA). Group comparisons for continuous data were performed using Student’s *t*-test or the Mann–Whitney U test, based on normality. Categorical variables, such as ED incidence at each time point, were analyzed using the χ^2^ test or Fisher’s exact test. Odds ratios (ORs) and 95% confidence intervals (CIs) were calculated. Statistical processing was conducted using SPSS software (version 31.0; IBM Corp., Armonk, NY, USA), with significance defined as *p*-value < 0.05.

## 3. Results

### 3.1. Characteristics of the Sample

**Figure 1:**
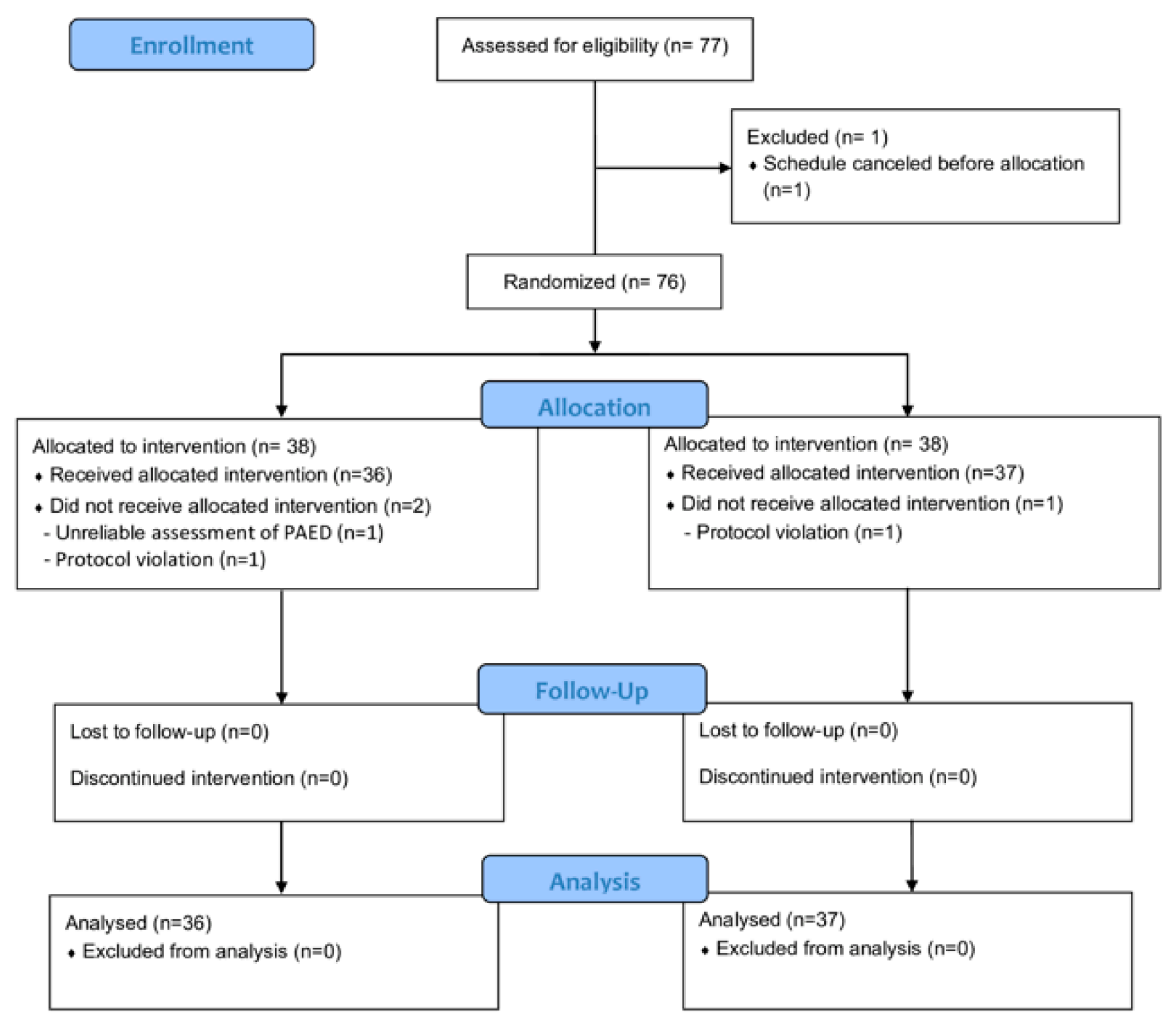
Flow diagram of patients’ enrollment.

A total of 77 patients were assessed for eligibility, of whom 76 were enrolled. One patient was excluded prior to allocation due to withdrawal of consent. During recruitment, three additional patients were excluded: two from Group P (one due to unreliable PAED assessment and one because no reversal agent was administered) and one from Group S (due to an allocation error). Ultimately, 73 patients (36 in Group P and 37 in Group S) were included in the analysis (Figure 1).

Baseline characteristics, including patient demographics, procedural details, and anesthetic management, were comparable between the two groups (Table 2).

### 3.2. PAED Scores at PACU

The incidence of ED was higher in Group P (50.0%) than in Group S (40.5%); however, this difference was not statistically significant (OR = 0.682, 95% CI = 0.270–1.721, *p* = 0.417). Similarly, the PAED scores at each time point (T1, T2, and T3) showed no significant differences between the groups. The peak PAED scores were 10.0 (7.0, 15.5) and 9.0 (7.5, 11.5) for groups P and S, respectively (*p* = 0.277). The additional requirement for rescue fentanyl doses was 19.4% in Group P and 13.5% in Group S (OR = 0.647, 95% CI = 0.185–2.266, *p* = 0.494). Additionally, there was no significant difference between the groups regarding the duration of PACU stay (49.1 ± 12.5 min in Group P vs. 49.5 ± 13.1 min in Group S; *p* = 0.893). (Table 3).

**Figure 2:**
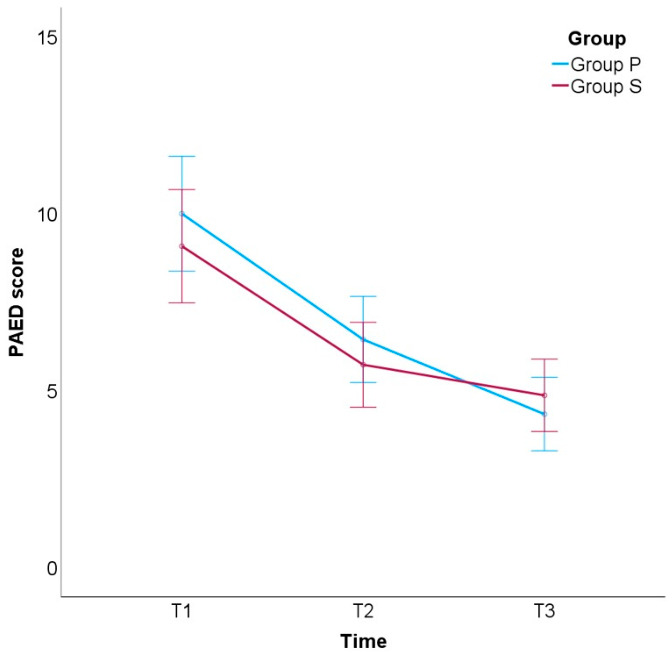
PAED score variations. Each blue and red dot indicates the PAED score at each time point of measurement. T1, upon PACU arrival; T2, 15 min after PACU arrival; T3, 30 min after PACU arrival. PAED, pediatric anesthesia emergence delirium.

RM ANOVA revealed that neither the overall intergroup difference in PAED scores (*p* = 0.629) nor the group-by-time interaction (*p* = 0.243) was significant. However, overall PAED scores significantly decreased compared to baseline values at T1 (*p* < 0.001) (Figure 2).

## 4. Discussion

This study demonstrated that the choice of reversal agent does not significantly affect the incidence of ED following pediatric strabismus surgery. The overall incidence of ED in PACU after strabismus surgery was 45.2%, with 50.0% observed in Group P and 40.5% in Group S. Although the peak PAED scores and the additional requirement for rescue doses were higher in Group P, these differences between the groups did not reach statistical significance. Consequently, the type of reversal agent appears to have no substantial impact on mitigating the severity of ED in this population.

Although this study found that sugammadex presented a lower incidence of ED compared with pyridostigmine, its effect on ED is not confirmatory. Sugammadex acts as a reversal agent by selectively binding to and encapsulating neuromuscular blocking agents, and this compound is biologically inactive. Sugammadex is superior to ChEI in reversing the residual NMB; hence, it is thought to be effective in mitigating ED [19,27]. Conversely, sugammadex did not reduce the incidence of ED in adult patients [28,29]. Interestingly, a recent study reported an association between sugammadex and early postoperative delirium (POD, occurring within 24 h post-extubation), suggesting that this early POD is phenotypically indistinguishable from ED [16]. There is no mechanism that could explain how sugammadex increases the incidence of early POD; it might be associated with unmeasured confounding factors [30].

Substantial evidence indicates that cholinergic system dysfunction is highly associated with the development of neurodegenerative diseases [31,32]. Reduced acetylcholine levels in the cortex and hippocampus impair cerebral activation mechanisms, leading to progressive cognitive decline and behavioral disturbances [31,33]. In this context, the administration of ChEI is considered to improve the cognitive function of patients with neurodegenerative diseases by enhancing cholinergic neurotransmission in the brain [16,34]. However, several reviews have reported that current evidence remains insufficient to support the use of ChEI for the prevention or treatment of delirium [35,36]. Furthermore, Rössler et al. and Batistaki et al. concluded that there is no significant difference in the incidence of postoperative cognitive dysfunction between neostigmine and sugammadex [16,37], findings that are consistent with the results of this study. Glycopyrrolate is a typical muscarinic antagonist; by inhibiting central and peripheral cholinergic transmission, it can increase the anticholinergic burden, leading to cognitive impairment and elevated risk of delirium [38]. Theoretically, ChEI and glycopyrrolate are known to be unable to cross the blood–brain barrier (BBB); however, a recent study reported that perioperative inflammation and the stress response make it easier to increase BBB permeability and facilitate passage of these agents and thereby elevate delirium risk [39].

Four potential explanations may account for the unexpectedly low incidence and severity of ED observed in the Group P. First, as Ieong et al. concluded in a meta-analysis, the impact of anticholinergic burden on delirium may be exacerbated by advancing age [40]. Therefore, the lower-than-expected incidence and severity of ED following pyridostigmine-glycopyrrolate administration in this study may suggest a different, perhaps lower, susceptibility within the pediatric population. Second, the average TOF ratio at the time of reversal agent administration in Group P was 58%, and no additional rocuronium was required after intubation in any of the patients. Consequently, according to our protocol, a lower dose of glycopyrrolate was administered to the majority of the study population, which may have resulted in a lower-than-expected anticholinergic effect. Had a higher dose of ChEI and glycopyrrolate been administered, the incidence and severity of ED might have differed. Third, the sugammadex dosage used in this study may have had a limited impact on the prevention of ED. Several studies have indicated that the dose of sugammadex required to reverse NMB in pediatric patients may be higher than in adults, owing to immature neuromuscular transmission and a heightened sensitivity to neuromuscular blocking agents [41,42]. Consequently, it is possible that the sugammadex dose in the present study was insufficient to effectively mitigate ED. Fourth, physiological differences in delirium between adult and pediatric patients might account for the comparable results observed in the present study. POD in adults and ED in children share some phenomenological similarities, but they are regarded as distinct entities with different pathophysiological processes. In older adults, the pathogenesis of POD is thought to arise from neuroinflammation with microglial activation, increased BBB permeability, cholinergic deficiency, imbalances in dopaminergic and glutamatergic transmission, as well as a mismatch between cerebral metabolic demand and oxygen delivery [35]. Accordingly, alterations in cholinergic activity are considered to play a pivotal role in the development of POD in elderly patients. In contrast to adult patients, ED in pediatric patients has been closely linked to the pharmacodynamic and pharmacokinetic properties of volatile anesthetics in the context of an immature and developing brain. Hypothesized mechanisms include the rapid washout of volatile agents, which may lead to disorganized cortical activity, age-dependent differences in GABAergic and glutamatergic signaling, and transient sensory mismatch or dysphoria during reorientation [43]. Consequently, the influence of ChEIs on ED in pediatric patients appears to be limited; indeed, clinical trials to date have failed to demonstrate a clear benefit of conventional ChEIs for preventing or treating ED in this population.

Pediatric strabismus surgery is a well-established risk factor for ED, with reported incidence rates as high as 40–86% [44,45,46]. Compared with the previous two studies involving pediatric ophthalmologic surgery, which reported overall incidences of 67.7% and 67.4%, the incidence of ED in the present study was relatively low. This discrepancy may be explained by the longer operative duration in our cohort [47,48]. While the aforementioned studies reported average procedure durations of less than 20 min, the mean duration in our study was approximately 40. A shorter procedural duration has been previously suggested to correlate with a higher incidence of ED [49,50].

There are several limitations to the present study. First, pyridostigmine was utilized instead of neostigmine because the latter was unavailable at our center due to a nationwide supply shortage in South Korea. However, as pyridostigmine is a structural analog of neostigmine with similar lipid solubility, it is reasonable to assume that both agents would yield similar clinical outcomes in the context of this study. Second, postoperative pain was not formally assessed in this study, as we anticipated that pediatric patients would find it challenging to distinguish between surgical pain and ocular irritation caused by the application of ointment. Nevertheless, evaluating postoperative pain might have provided further evidence to elucidate the relationship between NMB reversal agents and ED more clearly. Finally, late recurarization during the early PACU period was not monitored. Because TOF stimulation is inherently painful, applying it to awake pediatric patients was deemed unethical. Additionally, quantitative TOF monitoring devices were unavailable in our PACU. To ensure a stable recovery profile suitable for extubation, the TOF ratio was measured for 5 min at 20 s intervals once the ratio initially reached 0.9. Although late recurarization in the PACU cannot be objectively excluded, we minimized this risk through meticulous, sustained TOF monitoring prior to extubation.

## 5. Conclusions

In conclusion, the type of NMB reversal agent was not significantly associated with the incidence of ED in the preschool-aged, pediatric population undergoing strabismus surgery. Despite the high-risk age group, the relatively long duration of the procedure may have contributed to the overall low incidence of ED observed. Further investigation involving deeper levels of NMB is warranted; in such cases, sugammadex may demonstrate a more favorable effect in preventing ED.

## Figures and Tables

**Table 1:** Pediatric anesthesia emergence delirium (PAED) scale.

Item
The child makes eye contact with the caregiver The child’s actions are purposeful The child is aware of his/her surroundings The child is restless The child is inconsolable

Items 1, 2, and 3 are scored: 4 = not at all, 3 = just a little, 2 = quite a bit, 1 = very much, 0 = extremely. Items 4 and 5 are scored: 0 = not at all, 1 = just a little, 2 = quite a bit, 3 = very much, 4 = extremely.

**Table 2:** Characteristics of patients, procedure, and anesthesia.

	Group P (*n* = 36)	Group S (*n* = 37)	*p* Value
Age (yr)	6.0 (5.0, 6.0)	5.0 (4.0–6.0)	0.635
Male, *n* (%)	12 (33.3)	19 (51.4)	0.119
Height (cm)	112.3 ± 9.9	112.4 ± 11.2	0.982
Weight (kg)	18.7 (17.2, 23.3)	21.1 (17.0, 22.7)	0.651
ASA (I/II), *n*	35/1	36/1	0.984
Mean EtSevo concentration (vol%)	1.0 (1.0, 2.0)	1.0 (1.0, 2.0)	0.932
Mean BIS	53.1 ± 5.9	53.0 ± 6.9	0.978
Mean BP (mmHg)	65.5 (57.5, 68.0)	64.0 (57.0, 71.0)	0.459
Mean HR (bpm)	93.8 ± 14.5	93.0 ± 12.9	0.803
Total infused remifentanil (μg)	199.0 (162.5, 256.5)	215.4 (176.0, 256.5)	0.351
TOF ratio, reverse (%)	58.2 ± 23.0	51.6 ± 30.3	0.322
TOF ratio, extubation (%)	97.7 ± 5.4	98.2 ± 5.4	0.712
Operation time (min)	40.0 (40.0, 50.0)	40.5 (36.3, 49.3)	0.854
Anesthesia time (min)	70.0 (65.0, 75.0)	70.0 (65.0, 78.5)	0.996

Values are expressed as mean ± SD, median (IQR) or number (%). ‘TOF ratio, reverse’ indicates TOF ratio value at the time of reversal agent administration. ‘TOF ratio, extubation’ indicates TOF ratio value at the time of extubation. ASA, American Society of Anesthesiologists physical status; BIS, bispectral index; BP, blood pressure; bpm, beats per minute; EtSevo, end-tidal sevoflurane; HR, heart rate; TOF, train-of-four.

**Table 3:** Postoperative ED profile at PACU.

	Group P (*n* = 36)	Group S (*n* = 37)	*p* Value	OR (95% CI)
Incidence of ED, *n* (%)	18 (50.0)	15 (40.5)	0.417	0.682 (0.270–1.721)
T1, *n* (%)	17 (47.2)	14 (37.8)	0.417	0.680 (0.268–1.729)
T2, *n* (%)	8 (22.2)	4 (10.8)	0.188	0.424 (0.115–1.559)
T3, *n* (%)	2 (5.6)	5 (13.5)	0.248	2.656 (0.481–14.678)
PAED score				
T1	9.0 (8)	9.0 (4)	0.409	
T2	6.4 ± 4.1	5.7 ± 3.2	0.429	
T3	4.0 (5)	6.0 (6)	0.492	
Peak PAED score	10.0 (9)	9.0 (4)	0.277	
Requirement for additional rescue dose, *n* (%)	7 (19.4)	5 (13.5)	0.494	0.647 (0.185–2.266)
PACU time (min)	49.1 ± 12.5	49.5 ± 13.1	0.893	

Data are expressed as mean ± SD, median (IQR), or number (%). T1, upon PACU arrival; T2, 15 min after T1; T3, 30 min after T1. CI, confidence interval; ED, emergence delirium; OR, odds ratio; PACU, post-anesthesia care unit; PAED, pediatric anesthesia emergence delirium.

## Data Availability

The data presented in this study are available upon request from the corresponding author due to restrictions imposed by the Institutional Review Board, which approved the study protocol.
